# Prevalence of acute critical neurological disease in children: a global epidemiological assessment (PANGEA)

**DOI:** 10.1186/cc12284

**Published:** 2013-03-19

**Authors:** E Fink, R Tasker, J Beca, MJ Bell, RS Clark, J Hutchison, M Vavilala, RS Watson, L Weissfeld, PM Kochanek, DC Angus, PANGEA Investigators

**Affiliations:** 1 Children's Hospital of Pittsburgh, PA, USA; 2 Children's Hospital Boston, MA, USA; 3 Starship Children's Hospital, Auckland, New Zealand; 4 The Hospital for Sick Children, Toronto, Canada; 5 Seattle Children's Hospital, Seattle, WA, USA; 6 University of Pittsburgh, PA, USA; 7 University of Pittsburgh Medical Center, Pittsburgh, PA, USA

## Introduction

Acute neurological injury is a leading cause of morbidity and mortality in children. Global prevalence and regional disparities of etiology, interventions, and outcomes are unknown. The aim of this point-prevalence study was to measure the burden of pediatric neurological injury and to describe variations in interventions and outcomes in ICUs.

## Methods

One hundred and three ICUs on six continents enrolled subjects on 4 specific days in a 1-year period. Included subjects were between ages 7 days and 17 years who were diagnosed with acute traumatic brain injury, stroke, cardiac arrest, central nervous system infection or inflammation, status epilepticus, spinal cord lesion, hydrocephalus, or brain mass. Sites completed a secure web-based case report form that included subject and hospital demographics, details about the neurological disease, interventions, length of stay, and Pediatric Cerebral Performance Category (PCPC) score (good outcome = PCPC 1 to 3) and mortality at hospital discharge.

## Results

Of 3,113 subjects screened, 1,009 (32%) met enrollment criteria. The mean number of subjects enrolled per site for each study day was 2.4. Most sites were dedicated pediatric ICUs with a mean number of 22 ICU beds (range 3 to 72). ICUs had resources to invasively monitor intracranial pressure (93%), continuous electroencephalography (14%), invasive and non-invasive brain tissue oxygenation (14% and 57%), and somatosensory evoked potentials (39%). There were on average 11 ICU faculty and six fellows per site, and nearly one-half reported a neurocritical care ICU team. Subjects were 58% male and 52% white, and 60% had normal pre-admission PCPC scores (85%). Status epilepticus and cardiac arrest (both 21%) had the highest prevalence. Sixty-one per cent of subjects were mechanically ventilated during ICU admission. ICU length of stay was a mean 29 days (median 43 days) and hospital LOS was a mean 43 days (median 61 days). Survival at hospital discharge was 87% with 58% of subjects discharged home and 17% to inpatient rehabilitation.

## Conclusion

Acute neurological disease is a significant pediatric health issue. These data suggest a vital need for increased research and healthcare resources to assist in the challenge of improving outcomes for these children.

